# Organ Specific Differences in Alteration of Aquaporin Expression in Rats Treated with Sennoside A, Senna Anthraquinones and Rhubarb Anthraquinones

**DOI:** 10.3390/ijms22158026

**Published:** 2021-07-27

**Authors:** Zhaoyang Wang, Ying Cheng, Wenting Su, Hongxia Zhang, Chu Li, Michael N. Routledge, Yunyun Gong, Boling Qiao

**Affiliations:** 1 Key Laboratory of Resource Biology and Modern Biotechnology in Western China, Ministry of Education, Northwest University, No. 229 TaiBai North Road, Xi’an 710069, China; wzy17864190778@163.com (Z.W.); 201820948@stumail.nwu.edu.cn (Y.C.); swt111354@163.com (W.S.); zth18942351323@163.com (H.Z.); jizhigirl@163.com (C.L.); 2 School of Medicine, University of Leeds, Leeds LS2 9JT, UK; 3 School of Food and Biological Engineering, Jiangsu University, Zhenjiang 212013, China; 4 School of Food Science and Nutrition, University of Leeds, Leeds LS2 9JT, UK; y.gong@leeds.ac.uk

**Keywords:** senna anthraquinones, rhubarb anthraquinones, sennoside A, laxative, aquaporins

## Abstract

Senna and rhubarb are often used as routine laxatives, but there are differences in mechanism of action and potential side effects. Here, we studied metabolites of senna anthraquinones (SAQ), rhubarb anthraquinones (RAQ) and their chemical marker, sennoside A (SA), in a rat diarrhea model. In in vitro biotransformation experiments, SAQ, RAQ and SA were incubated with rat fecal flora solution and the metabolites produced were analyzed using HPLC. In in vivo studies, the same compounds were investigated for purgation induction, with measurement of histopathology and *Aqps* gene expression in six organs. The results indicated that SAQ and RAQ had similar principal constituents but could be degraded into different metabolites. A similar profile of *Aqps* down-regulation for all compounds was seen in the colon, suggesting a similar mechanism of action for purgation. However, in the kidneys and livers of the diarrhea-rats, down-regulation of *Aqps* was found in the RAQ-rats whereas up-regulation of *Aqps* was seen in the SAQ-rats. Furthermore, the RAQ-rats showed lower Aqp2 protein expression in the kidneys, whilst the SA-rats and SAQ-rats had higher Aqp2 protein expression in the kidneys. This may have implications for side effects of SAQ or RAQ in patients with chronic kidney or liver diseases.

## 1. Introduction

Constipation is a common gastrointestinal disorder that affects 16% of the adult population globally [1]. Senna (*Cassia angustifolia* Vahl) and rhubarb (*Rheum* spp.) are widely used herbal laxatives, due to their rapid purgative actions and availability without prescription [2]. Senna and rhubarb are classified as stimulative laxatives, and known to have similar effective constituents, the anthraquinones [3]. The anthraquinones belong to anthranoid glycosides, for which sennoside A is used as a chemical marker for quality control [4,5]. Being protected by the β-glucoside bond, the anthraquinones can reach the intestines where they are degraded into complex metabolites by enzymes secreted from the intestinal microbiome [6,7]. The purgative action of sennoside A conversion to rheinanthrone in the intestine has previously been explained in terms of Na^+^/K^+^ transport, water and mucus secretion in the colon [8]. It is these complex metabolites that produce the laxative effects. The similarities and differences of action between anthraquinones require further elucidation.

In recent decades, alterations of aquaporins (AQPs) expression have been shown to be associated with constipation and diarrhea [9,10]. AQPs, a large family of integral membrane proteins, form pores that function as regulators of intracellular and intercellular water flow [11]. Thirteen known types of aquaporins (AQP0–AQP12) in mammals are classified into three main subfamilies in terms of their functional characteristics. They are water-specific channels (AQP0, AQP1, AQP2, AQP4, AQP5, AQP6 and AQP8), aquaglyceroporins (AQP3, AQP7, AQP9 and AQP10) and superaquaporins (AQP11 and AQP12) [12,13]. These AQPs are permeable to water and/or other small uncharged molecules such as ammonia, urea and glycerol. They are known to be differentially co-expressed in cells and various organs including colon, kidney, liver, lung, stomach and spleen. Alteration of the AQP expression levels could mediate the bidirectional flow of water and maintain homeostasis in the organs. It has been shown that no single AQP isoform is exclusively expressed at any single site [14]. To date, at least 11 AQPs have been found to be present in the small and large intestines [15]. Five and seven AQPs have been detected in human and mouse livers, respectively, as well as in the kidneys [16,17]. These AQP sub-types play key roles in water and fluid homeostasis. Various data indicate that AQP expression changes can be associated with constipation or diarrhea [8]. It has been reported that the laxative effect of the anthraquinones is associated with *Aqp3* alteration in the colon. Understanding the overall AQP changes would provide insight into the potential influence of the complex metabolites on the colon and even on other organs.

In this study, senna anthraquinones (SAQ) and rhubarb anthraquinones (RAQ) were prepared, and analyzed for the content of sennoside A (SA) by high performance liquid chromatography (HPLC). For comparison of similarities and differences between SAQ and RAQ, we performed in vitro biotransformation with rat fecal bacteria solution to determine metabolites, and in vivo rat studies to measure clinical signs, organ index (organ weight/body weight), pathological changes and *Aqps* expression profiles on six organs (colon, liver, kidneys, spleen, stomach and lungs), following induction of diarrhea with SAQ or RAQ. Using real-time quantitative reverse transcription polymerase chain reaction (RT-qPCR), *Aqp* alteration profiles were constructed and analyzed for each organ in ten subtypes including *Aqp1*, *Aqp2*, *Aqp3*, *Aqp4*, *Aqp5*, *Aqp6*, *Aqp7*, *Aqp8*, *Aqp9* and *Aqp11*. Furthermore, the Aqp2 protein expressions were examined in the rat kidneys using immunohistochemistry.

## 2. Results

### 2.1. Different Metabolite Profiles Produced from SAQ, RAQ and SA

**Figure 1:**
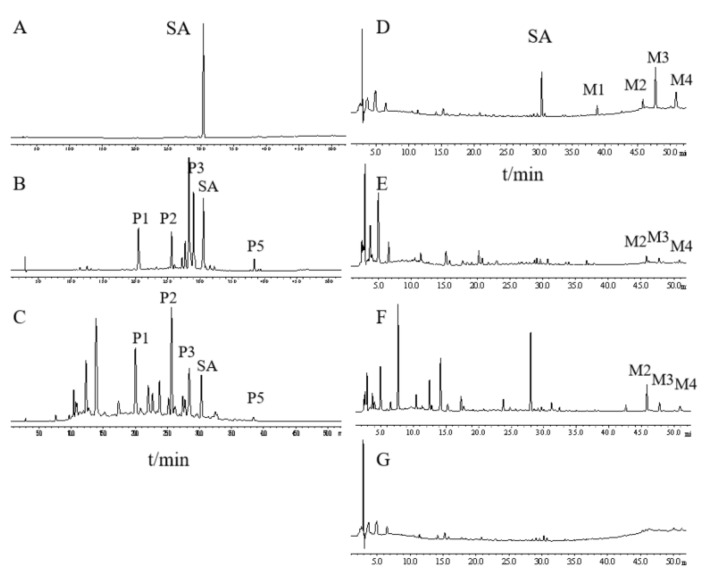
In vitro biotransformation was performed on the products in the rat fecal flora solution for 8 h, and metabolites produced were analyzed using HPLC. HPLC chromatogram of (**A**) sennoside A (SA). (**B**) senna anthraquinones (SAQ). (**C**) rhubarb anthraquinones (RAQ). (**D**) metabolites of SA. (**E**) metabolites of SAQ. (**F**) metabolites of RAQ. (**G**) the rat fecal flora solution.

To compare their chemical constituents, we performed HPLC analysis on SA (Figure 1A), SAQ (Figure 1B) and RAQ (Figure 1C).

Under the conditions used, the multiple components of the SAQ and RAQ could be clearly separated. Based on the standard sample, SA was identified and could be observed in both the SAQ and RAQ chromatograms. Five principal peaks (P1, P2, P3, P5 and SA) were involved in both anthraquinones suggesting similar parental constituents. In terms of peak area, SA content was determined as 8.13% (*w*/*w*) in SAQ and 4.57% (*w*/*w*) in RAQ.

It is known that the anthraquinones’ purgative actions are mediated through the metabolites produced in the intestine. To mimic the in vivo process, we performed in vitro biotransformation on RAQ, SAQ and SA by incubation in rat fecal bacteria flora solution (RFS). The biotransformation was conducted for 8 h and the metabolites produced (M-RAQ, M-SAQ and M-SA) were analyzed by the HPLC.

SA was successfully degraded, as indicated by the decreased SA peak and the additional four peaks (M1, M2, M3, and M4) shown in the M-SA chromatogram (Figure 1D). Based on the literature and standard samples, M1 was identified as emodin, M3 as rhein and M4 as aloe-emodin. Similarly, the peaks of the parental constituents shown in SAQ and RAQ disappeared and new peaks were found in the HPLC chromatogram of M-SAQ (Figure 1E) and M-RAQ (Figure 1F). The three new metabolites (M2, M3 and M4) produced from SA could also be found in M-SAQ and M-RAQ. However, other additional peaks displaying different retention times were evident in the HPLC profiles for M-SAQ and M-RAQ. Under the detection conditions used, no obvious peaks were shown in the RFS chromatogram (Figure 1G). Thus, by using in vitro transformation, we found that the three products yielded different mixtures of metabolites, which may have different actions in the body.

### 2.2. The Growth of Diarrhea-Rats Induced by SAQ, RAQ and SA

Using the SAQ, RAQ and SA, we generated diarrhea-rats with low- and high-grade diarrhea. The single compound SA, low dose of SAQ (L-SAQ) and RAQ (L-RAQ) resulted in diarrhea with loose stool (grade 1), and high dose of SAQ (H-SAQ) and RAQ (H-RAQ) resulted in watery diarrhea (grade 2). The single compound SA at 50 mg/kg could achieve diarrhea grade 1 only.

**Figure 2:**
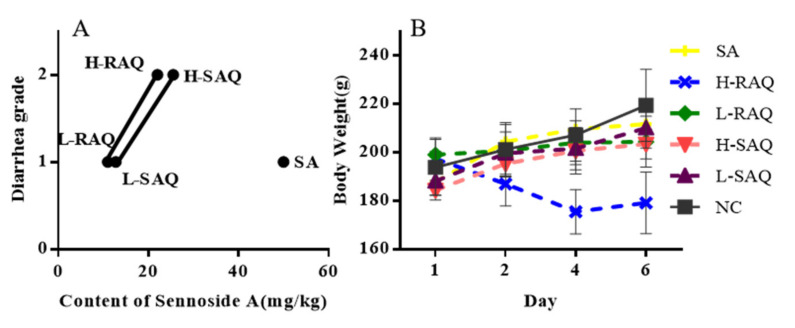
The male SD rats were treated without (NC) or with senna anthraquinones at low (L-Scheme 6 days). (**A**) SA content and diarrhea grade of the rats (1, 2 refers to loose stool and watery diarrhea, respectively). (**B**) The transition of body weight for the experimental rats.

SA content was determined to be 12.76 mg/kg and 25.52 mg/kg in L-SAQ and H-SAQ, respectively; and was 11.01 mg/kg and 22.03 mg/kg in L-RAQ and H-RAQ, respectively. Therefore, SAQ and RAQ contained lower SA concentrations than the 50 mg/kg SA that produced only grade 1 diarrhea, but showed more potent purgative action. This indicates that other constituents do contribute to the purgative function of SAQ and RAQ by targeting multiple genes. The rat diarrhea grade along with their SA content is shown in Figure 2A.

During the experiment, the body weight and clinical signs, including behavior and diet of the rats, were regularly recorded. The normal control group (NC-rats) exhibited normal growth rate of 13.2% until the last day of the experiments. The diarrhea-rats induced by SA, L-SAQ and H-SAQ showed a similar growth rate of 12.8%, 11.1% and 10.3%, respectively. The L-RAQ treatment resulted in the rats having slow growth with a rate of 4.0% (*p* = 0.0003) (In Figure 2B, the green line is horizontal). The rats in the H-RAQ group had significant reduction of mean body weight with a growth rate of −9.3% (Figure 2B).

### 2.3. Organ Index in the Diarrhea-Rats Induced by Senna Anthraquinones (SAQ), Rhubarb Anthraquinones (RAQ) and Sennoside A (SA)

**Figure 3:**
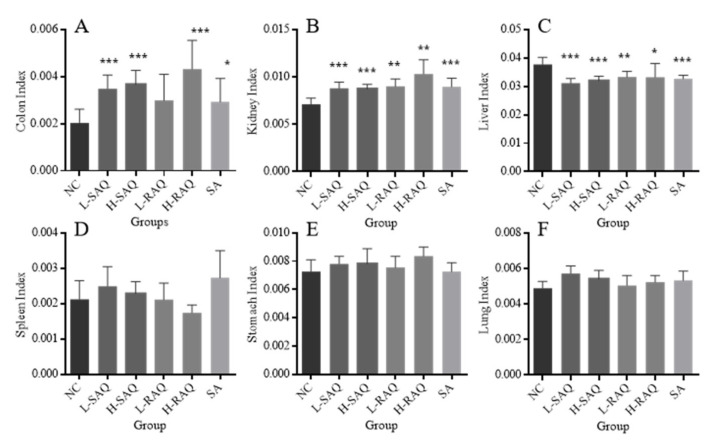
The organ index in normal control (NC) rats and diarrhea rats induced by a low dose of senna anthraquinones (L-SAQ), high dose of senna anthraquinones (H-SAQ), low dose of rhubarb anthraquinones (L-RAQ), high dose of rhubarb anthraquinones (H-RAQ) and sennoside A (SA) for over 6 days. * represents significant difference with the *p* < 0.05, ** represents significant difference with the *p* < 0.01, *** represents significant difference with the *p* < 0.001. (**A**) the colon index. (**B**) the kidney index. (**C**) the liver index. (**D**) the spleen index. (**E**) the stomach index. (**F**) the lung index.

Figure 3 describes organ index (colon, stomach, lungs, kidneys, spleen and liver) of the rats involved in the normal control rats (NC-rats) and diarrhea-rats groups.

All the treatments resulted in a significant increase for colon index, excluding L-RAQ (Figure 3A). The significant increase for both low and high SAQ groups and H-RAQ was over two times higher than the NC group (*p* < 0.0001). Relatively lower increases were found in L-RAQ and SA with the increase rate at 46.5% (*p* = 0.053) and 43.6% (*p* = 0.047), respectively.

An increase was seen in kidney index for all diarrhea-rats (Figure 3B), whilst there was a significant decrease in liver index in all diarrhea groups (Figure 3C). No significant difference was found in organ index for the spleen (Figure 3D), stomach (Figure 3E) or lungs (Figure 3F) between the NC-rats and all the diarrhea-rats.

Overall, the metabolites of the three products might be distributed into the organs (colons, kidneys and livers), indicated by the decrease in the rat liver index and increase in the rat colon index and kidney index. This suggested that the products had potential side effects on the liver and kidney.

### 2.4. Histopathological Changes in Colons Collected from the Diarrhea-Rats Induced by Senna Anthraquinones (SAQ), Rhubarb Anthraquinones (RAQ) and Sennoside A (SA)

**Figure 4:**
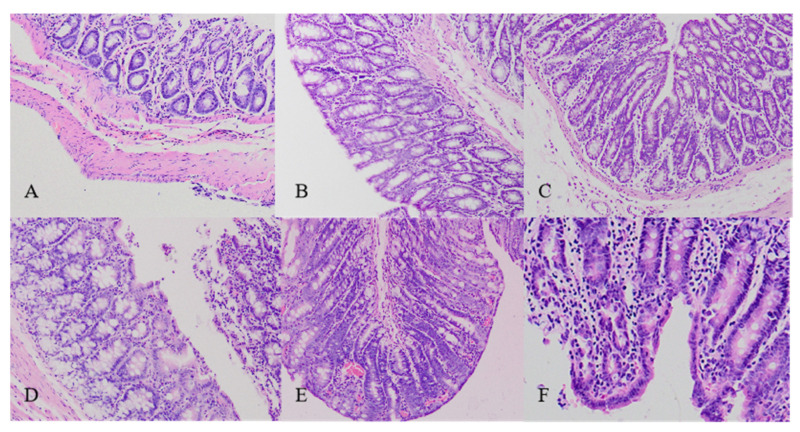
The H&E staining on the colons collected from the untreated rats and treated rats for over 6 days (original magnification 40×). The colon from (**A**) normal control rats. Rats treated with (**B**) low dose of senna anthraquinones. (**C**) high dose of senna anthraquinones. (**D**) low dose of rhubarb anthraquinones. (**E**) high dose of rhubarb anthraquinones. (**F**) sennoside A.

Histopathological changes were observed following H&E staining of paraffin-embedded colon tissue (Figure 4) but for none of the other five organs.

In the NC-rats, colons displayed intact epithelium and mucosa, no disruption on crypt architecture, complete goblet cells with mucus-filled vacuoles, and no infiltration of leukocytes (Figure 4A). However, moderate damage was found with decreased goblet cells in colons of the diarrhea-rats induced by low dose of the products including L-SAQ (Figure 4B) and L-RAQ (Figure 4D). Severe lesions were present with decreased goblet cells and inflammatory cell infiltration in colons of the diarrhea-rats induced by H-SAQ (Figure 4C) and H-RAQ (Figure 4E). Inflammatory cell infiltration in the crypt architecture was found in diarrhea-rats induced by SA treatment (Figure 4F).

The results indicated that the anthraquinone laxatives could damage the colon when overused or used for a longer time. It has been reported that patients with chronic use of anthraquinone-containing laxatives are prone to the development of melanosis coli [18].

### 2.5. The Aqps Alterations Shown in Diarrhea-Rats Induced by SAQ, RAQ and SA

Expression levels of ten subtypes of *Aqp*, including *Aqp1*, *Aqp2*, *Aqp3*, *Aqp4*, *Aqp5*, *Aqp6*, *Aqp7*, *Aqp8*, *Aqp9* and *Aqp11* in six organs were compared to the normal control (NC) group, with *Hprt* as a reference gene. The changes for each subtype shown in diarrhea-rats were calculated based on the following:

The fold change = (level in diarrhea-rats—level in NC-rats)/ level in NC-rats

As such, the fold change was a positive number for the up-regulated subtype and was a negative number for the down-regulated subtype. Then, the *Aqps* alteration profile for each organ could be figured as a heatmap.

On the other hand, to distinguish the differences among the treatments, PCA was applied for species discrimination and integrative quality evaluation. The PCA results could display distinct clusters among the groups including NC, L-SAQ, H-SAQ, L-RAQ, H-RAQ and SA. The similar *Aqps* alteration profiles would be aggregated together in one cluster. The influence of the treatment could be evaluated by the distance between the treated groups and the NC group. A farther distance implied a greater difference.

#### 2.5.1. Colon

**Figure 5:**
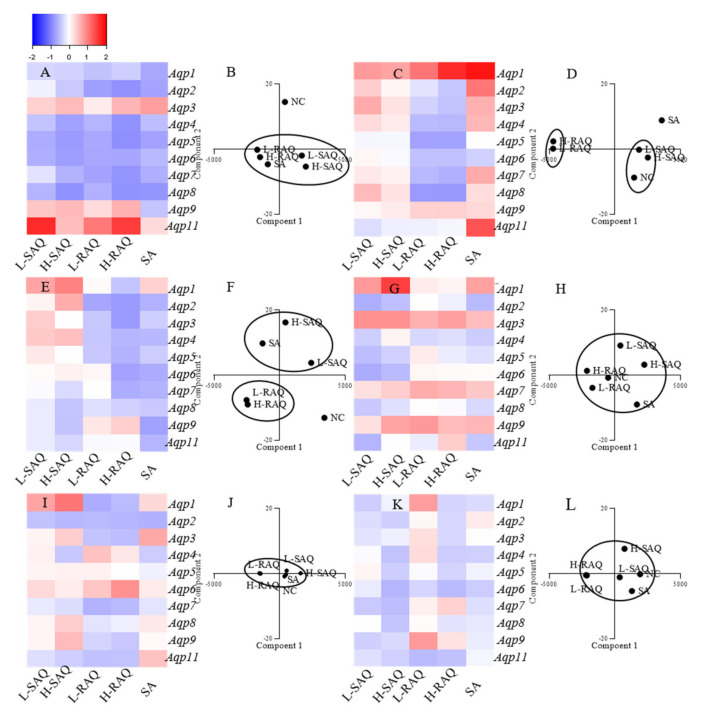
RT-qPCR was used to detect *Aqps* expression in each organ collected at day six from the control-rats (NC) and diarrhea-rats induced by senna anthraquinones at low (L-SAQ) and high dose (H-SAQ), rhubarb anthraquinones at low (L-RAQS) and high dose (H-RAQ), and sennoside A (SA). Heatmap with *Aqp* alteration profiles in the (**A**) colon. (**C**) kidney. (**E**) liver. (**G**) stomach. (**I**) lung. (**K**) spleen. Negative number represents the fold changes of down-regulated *Aqps*, and positive number represents the fold changes of up-regulated *Aqps*. PCA results for the (**B**) colon. (**D**) kidney. (**F**) liver. (**H**) stomach. (**J**) lung. (**L**) spleen.

Downregulation occurred in a dose-dependent manner for most of *Aqps* types in the rats treated with SAQ and RAQ (Figure 5A). The fold changes were over 0.5-fold for the L-SAQ group on *Aqp4*, *Aqp5*, *Aqp6, Aqp7* and *Aqp8*; in RAQ groups on *Aqp2*, *Aqp4*, *Aqp5*, *Aqp6, Aqp7* and *Aqp8*. The rats in the RAQ group showed the down regulation on *Aqp5* from 0.68-fold to 0.87-fold, on *Aqp6* from 0.68-fold to 0.69-fold, on *Aqp7* from 0.71-fold to 0.83-fold, and on *Aqp2*, *Aqp4* and *Aqp8* from 0.74-fold, 0.58-fold and 0.60-fold to 0.85-fold, respectively. The rats in SAQ showed down regulation on *Aqp8* from 0.57-fold to 0.86-fold, *Aqp5* from 0.64-fold to 0.78-fold, *Aqp6* from 0.62-fold to 0.76-fold and *Aqp4* from 0.46-fold to 0.74-fold changes. The rats in SA group also showed similar down-regulation on all the *Aqps* excluding *Aqp9*.

In the PCA results, there were clearly differences between the NC and diarrhea groups (Figure 5B). The SAQ groups on both are localized in the same cluster. The group SA and RAQ on both are in another cluster. However, the group NC is clearly separated in an individual cluster and is far away from all the diarrhea groups indicating similar laxative effects from all the products.

#### 2.5.2. Kidneys

There were opposite changes shown in group RAQ and others (Figure 5C). Minor changes (less than 0.5-times change) with slight up-regulations were found in SAQ on both groups, whereas down-regulations on most of the *Aqps* were the main characteristics for the RAQ-rats. The prominent down-regulations shown in group L-RAQ and H-RAQ were observed from 0.54-fold to 0.55-fold on *Aqp4*, from 0.71-fold to 0.72-fold on *Aqp5*, from 0.54-fold to 0.62-fold on *Aqp7*, from 0.79-fold to 0.80-fold on *Aqp5*. In the SA-rats, the obvious increase was found in most of *Aqps*.

In the PCA results (Figure 5D), the NC group and SAQ on both are localized in one cluster. The SA group and the group RAQ on both are clearly distinguished by different cluster, which are far away from the NC group.

The results suggested that RAQ had more different influences than RAQ.

#### 2.5.3. Liver

There were different *Aqp* alteration profiles among the diarrhea rats (Figure 5E). Down-regulation on most of the *Aqps* were the characteristics of the rats in group SA and RAQ. The prominent down regulation was shown in group SA on *Aqp9* with 0.74-fold changes, on *Aqp6* with 0.64-fold changes, on *Aqp2* and *Aqp8* with 0.60-fold changes. The down regulation observed in group L-RAQ and H-RAQ was from 0.70-fold to 0.77-fold changes on *Aqp2*; from 0.44-fold to 0.57-fold changes on *Aqp4*, from 0.42-fold to 0.60-fold changes on *Aqp5*, from 0.06-fold to 0.72-fold changes on *Aqp6*, and from 0.01-fold to 0.63-fold changes on *Aqp7*. The rats in group SAQ had minor changes with slight up- or down-regulation (less than 0.5-times) on most of the *Aqps*.

The PCA results (Figure 5F) show that the RAQ on both groups are localized in one cluster. The SA and SAQ on both groups are set in two clusters which are far away from the NC group. This implies that SA and RAQ had different actions on the livers although they all showed down-regulated *Aqps* profiles.

#### 2.5.4. Stomach

In the stomach (Figure 5G), no consistent alterations were shown on most of *Aqps* in SAQ and SA groups, and minor changes were observed in RAQ groups. In the PCA results, all the diarrhea groups are near the NC group, suggesting similarities among the groups (Figure 5H).

#### 2.5.5. Lungs

In the lungs (Figure 5I), no obvious differences were observed in the diarrhea-rats. The PCA results display aggregated points among the diarrhea groups and the NC group (Figure 5J).

#### 2.5.6. Spleen

For the spleen (Figure 5K), minor alterations (less than 0.5-times changes) on all the *Aqps* were observed in all the diarrhea-rats. The PCA results also show aggregate points without obvious differences between the diarrhea-rats and the NC-rats (Figure 5L).

Overall, in the diarrhea-rats, down-regulation was the characteristic profile of the colon (Table 1). In the kidneys, up-regulation was seen for the diarrhea-rats induced by SAQ and SA. Down-regulation was the characteristic profile of the kidney and liver in the rats treated with RAQ.

### 2.6. Aqp2 Protein Expressions in the Kidneys of Diarrhea-Rats Induced by Senna Anthraquinones (SAQ), Rhubarb Anthraquinones (RAQ) and Sennoside A (SA)

**Figure 6:**
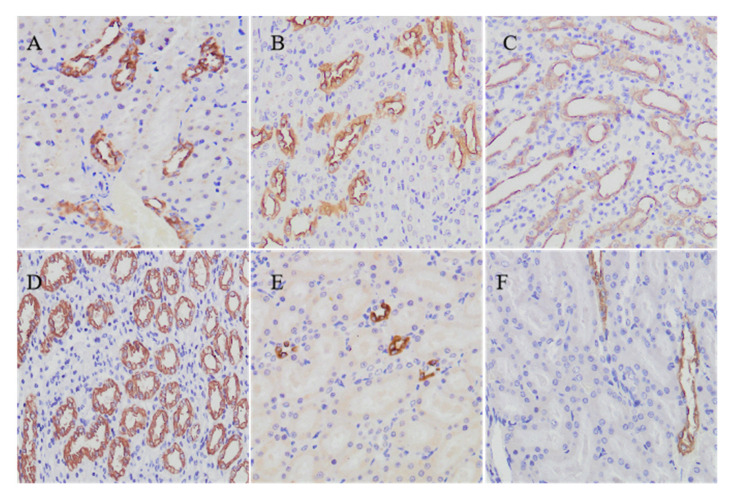
IHC analysis of Aqp2 on the kidneys collected at day six from (**A**) the normal control rats. Diarrhea-rats induced by (**B**) low dose of senna anthraquinones. (**C**) high dose of senna anthraquinones. (**D**) low dose of rhubarb anthraquinones. (**E**) high dose of rhubarb anthraquinones. (**F**) sennoside A (SA). Magnification: 200×.

In our study, Aqp2 could be detected in paraffin-embedded sections of rat kidney tissues by using immunohistochemistry. In the NC rats, Aqp2 labeling is seen in the apical plasma membrane and in the collecting duct principal cells (Figure 6A). Mainly in the medulla collecting duct, there were more cells labeled in the rats administered with L-SAQ (Figure 6B) and H-SAQ (Figure 6C). In SA rats, there was a marked increase in the Aqp2 immunolabelled cells compared to the NC rats (Figure 6D). In contrast, the rats administered with L-RAQ (Figure 6E) and H-RAQ (Figure 6F) revealed less immunolabelled cells in the medulla collecting duct. We also noticed that the RAQ-rats had more urine than the NC-rats, but the SAQ-rats and SA-rats showed no such phenomenon.

## 3. Discussion

In general, herbal drugs are orally administrated, so components are inevitably exposed to the intestinal flora. Consequently, the metabolites of such interactions are produced, absorbed by the gut and distributed into various organs. Thus, the pharmacological/toxic effects of the compounds are determined by the metabolites produced in the intestine. Our results showed that SAQ and RAQ had quite similar parental constituents but yielded different metabolites. We deduced that they may have different actions in the body. However, it is a challenge to assess their activities in terms of their complex metabolites.

In prokaryotes and eukaryotes, AQPs are differentially expressed in various organs. They are known to facilitate transmembrane water fluxes and transport of small solutes including glycerol, urea, CO_2_ and nitric oxide [19]. To date, AQP changes have been demonstrated to be associated with drug treatments and some diseases, because their alterations represent the disturbance of the small molecule and water transport leading to changes in the physiological intracellular and extracellular environment [20].

AQPs in the colon have been demonstrated to have important roles in many studies [15]. It is known that the colon epithelium absorbs about 1.5–2 L/day of water against an osmotic gradient, most of which moves through AQPs. Varying AQP expression levels have been observed in the intestinal tract in mammalian species, including AQP1, AQP2, AQP3, AQP4, AQP5, AQP6, AQP7, AQP8 and AQP9 [16,21]. The down regulation of AQPs could prevent water being transmitted into the cells, instead being reabsorbed in the colon, leading to a high water content in rat feces [22,23]. It is known that many laxative drugs produce purgative actions through down-regulating *Aqps* expression in the colon [24,25]. Our results demonstrated that down-regulating *Aqps* would be the main mechanism of SA, SAQ and RAQ leading to rapid purgation. Furthermore, the down-regulations were observed on multiple *Aqps* suggesting the cooperative effects coming from the complex metabolite components.

As the main organs of drug metabolism, the kidney and the liver are of particular relevance. In humans, over 150 L of aqueous fluid is filtered through the kidneys each day. At least eight AQPs are expressed in kidneys, and play a vital role in regulating water balance and small solutes [26,27]. In theory, down-regulation of the *Aqps* would prevent water/small solute flux into kidneys but flux into urine. For example, AQP7 is abundantly present at the apical membrane of the proximal straight tubules in the kidney. *Aqp7* null mice showed lower serum glycerol levels but marked glycerol in their urine, and significant increased urine volume [28]. AQP2 is abundantly expressed in the kidney cells along the connecting tubule and duct, where it is localized at the apical plasma membranes and membranes of intracellular vesicles [29]. It was reported that the urine volume was increased up to 1.5-fold in mice deleted for *Aqp2* gene specific to the mouse connecting tubules [30]. Our results showed that most of the *Aqps* expression in the kidneys was decreased in the RAQ-rats but increased in the SAQ-rats and SA-rats. The Aqp2 expression was lower in the RAQ-rats but higher in the SAQ-rats and SA-rats. We deduced that SA, SAQ and RAQ were distributed to the kidney with different metabolites resulting in opposite actions on the kidneys through modulating *Aqps*.

The liver plays a central role in glycerol metabolism, due to its major (70–90%) contribution to the whole-body glycerol metabolism [31]. Aquaglyceroporins (AQP3, AQP7 and AQP9) were detected in human liver, and are thought to play key roles in mediating glycerol transport in cells. It has been reported that adipose *Aqp7* and *Aqp9* gene expression was increased by diet-induced obesity in mice [32]. AQP3 was expressed in hepatocytes and Kupffer cells which are involved in pathogenesis of liver diseases [33]. Deficiency of *Aqps* was suggested to reduce glycerol influx into hepatocytes, thereby preventing the development of metabolic diseases such as obesity, diabetes, and non-alcoholic fatty liver diseases [32,33,34]. Our results showed that SA and RAQ could down regulate most of the *Aqps* in livers of the rats, particularly *Aqp3, Aqp7* and *Aqp9*. In addition, SA has been reported to protect mitochondrial function and structure to improve hepatic steatosis by inhibiting the mitochondrial respiratory chain complex I and the voltage-dependent anion channel 1 (VDAC1) [35]. Rhubarb extract has been reported to prevent hepatic inflammation induced by acute alcohol intake [36]. Assuming these effects may be due to *Aqps* down-regulation, patients with certain liver diseases might benefit from *Aqps* regulation.

However, it is also worth noticing that their usage should not be continued for the long-term. This could be harmful/toxic to the colon or other organs. Patients with chronic use of anthraquinone-containing laxatives have been shown to be prone to the development of melanosis coli [18]. We have suggested that the metabolites could be accumulated over time resulting in colon cell death [37]. Moreover, severe inflammation was found in our study in the colon of the rats administered with high doses for 6 days. Recently, it has been suggested that AQPs are involved in the development of inflammatory mechanisms, supported by the detection of several isoforms in cells of both the innate and adaptive immune system and the demonstration of their dysregulation in various human diseases. For example, AQP8 downregulation was described in human Crohn’s disease and ulcerative colitis biopsies [38]. Our study showed that drug induced *Aqp8* downregulation occurred in a dose dependent manner, which could lead to colon inflammation after long-term usage.

## 4. Materials and Methods

### 4.1. Reagents and Preparation of Senna Anthraquinones (SAQ) and Rhubarb Anthraquinones (RAQ)

Dried senna (*Cassia angustifolia* Vahl) leaves and rhubarb (*Rheum palmatum* L.) roots were purchased from Shaanxi Pharmaceutical Holding Group Co., Ltd., Xi’an, China. They were extracted and purified by using D101 Macroporous adsorption resin column (Sunresin Inc., Xi’an, China) according to our previously published method [37,39]. The content of sennoside A (SA) in the SAQ and RAQ were quantified by HPLC (see below).

SA was obtained from Weikeqi Bio-Tech Co. Ltd. (Chengdu, China). Methanol used was of HPLC grade and was obtained from Sigma-Aldrich (St. Louis, MO, USA). Purified water was produced using a reverse osmosis Milli-Q (18MΩ) system (Qingdao Flom Technology Co., Ltd., Qingdao, China).

### 4.2. HPLC Apparatus and Analytical Conditions

The HPLC apparatus consisted of a Shimadzu DHU-20A online degasser, two Shimadzu LC-20AD pumps and SPD-M20A Photo-diode array detector (Shimadzu, Tokyo, Japan). Chromatographic separations were achieved on an Hedera Si C18 column (4.6 × 250 mm, 5 μm, Hanbang Sci. & Tech, Huaian, China) at 35 °C and the detection wavelength was set at 280 nm, with a mobile phase consisting of methanol (A) and 0.1% phosphoric acid (B) at a flow rate of 1 mL/min. The gradient elution was run as follows: 10–27% A at 0–5 min; hold 27% A at 5–10 min; 27–53% A at 10–25 min; 53–80% A at 25–40 min and 80–80% A at 40–55 min.

### 4.3. Rats and Diarrhea Models Induced by the SAQ, RAQ and SA

Sixty male Sprague Dawley (SD) rats weighing 180–200 g were purchased from Dashuo experimental animal Co. Ltd. (Chengdu, China) (certificate No. SCXK-2014-028). Prior to the experiment, all animals were acclimatized in a pathogen-free-grade animal room under controlled conditions (24 ± 1.0 °C, 60 ± 5% humidity with a 12 h/12 h light-dark cycle) for three days and received standard laboratory chow and tap water ad libitum. All procedures for the care and handling of animals used in the study were approved in March of 2019 by the Ethics Committee of Northwest University for Animal Experimentation (ACCNU-2019-0013) and performed according to the Guidelines for Animal Experimentation of Northwest University and the National Institutes of Health guide for the care and use of Laboratory animals (NIH Publications No. 8023, revised 1978).

For the experiments, rats were randomly divided into six groups: normal control (NC), low dose SAQ (L-SAQ, 156.94 mg/kg), high dose SAQ (H-SAQ, 313.88 mg/kg), low dose RAQ (L-RAQ, 241.25 mg/kg), high dose RAQ (H-RAQ, 482.50 mg/kg) and SA (50 mg/kg), with 8 rats in each group. The rats were intragastrically administrated with 2.0 mL of drug solution or distilled water daily for each of six consecutive days. They were given free access to food and water throughout the study.

During the drug-dosing period, the rats were observed with body weight, stool consistency and behavior status recorded daily. Stool consistency was observed by the water content and set as three grades (grade 0, normal; grade 1, loose stool; grade 2, watery diarrhea). At day 6, rats were euthanized by intraperitoneal injection of ethyl carbamate (1 g/kg body weight) 4 h after the last administration, and organs including stomachs, spleens, livers, lungs, kidneys and colons were collected. The organs were weighed immediately followed further processing. Organs collected from three rats in each group were fixed in 4% paraformaldehyde to be embedded in paraffin. Organs from five rats were stored at −80 °C for mRNA extraction.

### 4.4. Hematoxylin and Eosin (H&E) Staining and Immunohistochemistry on Tissue Slides

The tissues embedded in paraffin were sectioned into 0.45 μm slices that were placed on glass slides (colon, lung, stomach, spleen, kidney and liver).

For H&E staining, the slides were deparaffinized and stained with hematoxylin and eosin. Following dehydration in alcohol and clearing in xylene, the slides were covered for imaging under a light microscope.

For immunohistochemistry, the section was dewaxed and heat mediated antigen retrieval performed in citrate buffer (pH6, epitope retrieval solution) for 15 min. The tissue section was blocked with 3% goat serum, and then incubated with rabbit anti-aquaporin 2 antibody (GB112259, diluted 1:600, Servicebio, Wuhan, China) overnight at 4 °C. Biotinylated goat anti-rabbit IgG (G1213, diluted 1:200, Servicebio, Wuhan, China) was used as secondary antibody and incubated for 30 min at 37 °C. The tissue section was developed using Strepavidin—Biotin—Complex with DAB (G1211, Servicebio, Wuhan, China) as the chromogen and covered for imaging under a light microscope.

### 4.5. Extraction of Total RNA from Rat Tissues and cDNA Synthesis

Tissues samples were homogenized in TRIzol reagent (Sangon Biotech Co., Ltd., Shanghai, China) using a tissue grinder (Ningbo Scientz Biotechnology Co., Ltd., Ningbo, China) at 4 °C. Total RNA was extracted using Trizol Reagent according to the manufacturer’s instructions. The concentration of RNA was determined by using a NanoDrop 1000 Spectrophotometer (GE Co., Boston, MA, USA). The quality of RNA was checked using the 260/280 nm ratio, with the values ranging from 1.8 to 2.0. All samples were subjected to cDNA synthesis under reverse transcriptase polymerase chain reaction (RT-PCR) by using random hexamer primers and TaqMan Reverse Transcription Reagents (Sangon Biotech Co., Ltd., Shanghai, China).

### 4.6. Primer Specificity and Real Time PCR

Various sets of gene-specific forward and reverse primers were designed by using a web-based tool (qPrimerDepot), as previously described [39]. Their specificities were demonstrated by the single bands of expected size in agarose gel electrophoresis and by the single-peak melting curves of the RT-qPCR products, which ranged from 100 to 300 bp.

The RT-qPCR was performed on cDNA (60 ng) after being mixed with SYBR Green Real-Time PCR Master Mix (Sangon Biotech Co., Ltd., Shanghai, China). All reactions were performed in triplicate in a final volume of 20 µL and analyzed with Bio-Rad real-time PCR system (CFX Connect, Bio-Rad Laboratories Inc., Hercules, CA, USA). The thermal cycle used was 10 min at 95 °C, denaturing for 15 s at 95 °C and annealing for 30 s at 60 °C. PCR amplification was performed for 40 cycles. Data are expressed as expressions relative to that of HPRT using 2^-∆∆Ct^ method. The mRNA levels are mean of values obtained from at least three rats in each group.

### 4.7. In Vitro Biotransformation of the SAQ, RAQ and SA

The rat feces were collected and processed immediately using a slightly modified method [40]. Briefly, the fresh feces were mixed with five-fold of sterile physiological saline, and the mixture was centrifuged at 3000 rpm for 10 min at 4 °C. The supernatant was incubated at 37 °C under anaerobic conditions for 8 h with the SAQ, RAQ or SA, respectively. The incubation was terminated by adding an equal volume of methanol to the culture solution, followed by centrifugation at 12,000 rpm for 10 min. The supernatant was filtered (0.22 µm pore size) and stored at −20 °C until HPLC was performed.

### 4.8. Heatmap, Principal Component Analysis (PCA) Analysis and Statistical Analysis

The heatmap was created using R program (version 3.5.1, Free Software Foundation, Inc., Boston, MA, USA). The PCA analysis was run using Past3 software. Statistical analyses were performed using SPSS 19.0 software and GraphPad Prism version 6.0 (GraphPad Software, Inc., San Diego, CA, USA). All results are expressed as means ± SD. Multiple comparisons were made by one-way ANOVA followed by Dunnett’s test. Results with *p <* 0.05 were considered to be statistically significant.

## 5. Conclusions

We demonstrated that SAQ and RAQ had similar principal constituents but could be degraded into different metabolites following the in vitro biotransformation. SAQ and RAQ showed similar laxative actions with a similar mechanism, indicated by the similar down-regulated *Aqps* profiles in the rat colon. In rat kidneys and liver, however, they could display different actions. The RAQ-rats had down-regulated *Aqps* profiles in the kidneys and liver, whereas the SAQ-rats displayed up-regulated *Aqps* profiles. Furthermore, immunohistochemistry on the rat kidneys revealed lower Aqp2 protein labeling cells in the RAQ-rats but higher Aqp2 protein labeling cells in the SA-rats and SAQ-rats. We suggest that the clinical usage of senna or rhubarb products should be clarified for patients having chronic kidney or liver diseases.

## Figures and Tables

**Table 1:** Summary of the *aqps* alterations for the three treatments.

Treatments	In the Colons *	In the Kidneys	In the Livers
SAQ	↘	↗	↗
RAQ	↘	↘	↘
SA	↘	↗	↘

* ↘ = down-regulated; ↗ = up regulated.

## Data Availability

Data is contained within the article.

## Abbreviations

Aqps	Aquaporins,
H&E staining	Hematoxylin and eosin staining,
HPLC	High performance liquid chromatography,
HPRT	hypoxanthine phosphoribosyltransferase,
IHC	Immunohistochemistry
NC	Normal control,
PCA	principal component analysis,
RFS	rat fecal bacteria flora solution
RAQ	Rhubarb anthraquinones,
RT-qPCR	Reverse transcription quantitative polymerase chain reaction,
SA	Sennoside A,
SAQ	Senna anthraquinones,
SD rat	Sprague Dawley rat,
RFS	Rat fecal flora solution,
